# Moving behavioral interventions in nursing homes from planning to action: a work system evaluation of a urinary tract infection toolkit implementation

**DOI:** 10.1186/s43058-023-00535-y

**Published:** 2023-12-12

**Authors:** James H. Ford, Anna T. Nora, Christopher J. Crnich

**Affiliations:** 1 Social and Administrative Sciences Division, University of Wisconsin School of Pharmacy, Madison, WI USA; 2 William S. Middleton VA Hospital, Madison, WI USA; 3 School of Medicine & Public Health, University of Wisconsin, Madison, WI USA

**Keywords:** Nursing homes, Urinary tract infections, Quality improvement, Change management, Implementation science, SEIPS, IMUNIFI, Planned versus actual changes, Coach notes

## Abstract

**Background:**

Implementation evaluations based on a hybrid deductive-inductive approach provide a detailed understanding of organizational choices to introduce and implement complex interventions and may help explain implementation success or failure. However, such evaluations may not be feasible due to resource constraints. Qualitative analyses of artifacts collected for other purposes during implementation may represent a cost-effective method to understand program implementation when robust evaluations are not feasible. This study used a work systems evaluation of how nursing homes (NHs) implemented a urinary tract infection (UTI) recognition and management improvement toolkit.

**Methods:**

Thirty NHs participated in a randomized control trial in which intervention NHs (*n* = 12) were assigned a clinical coach who employed a standard template to structure coach calls with the NH champion. A hybrid inductive-deductive approach, using the Systems Engineering Initiative for Patient Safety (SEIPS) model, characterized three action domains related to (1) engagement of staff and providers, (2) distribution of toolkit elements, and (3) toolkit use.

**Results:**

A total of 369 coded segments from 148 coach notes generated by three coaches working with 18 NH champions were examined. Planned changes (*n* = 203) were more frequent compared to actual changes (*n* = 169). While most NHs quickly engaged staff and providers, which leadership appeared to support, engagement actions were hindered in some NHs due to champion instability or extended champion or medical director absences. Dissemination of materials to family and providers and distribution of tools to staff occurred quickly in 75% of NHs, although delays were encountered in some NHs, usually because of champion instability.

**Conclusions:**

Implementing NH practice change is challenging, and studies examining actions to support planned versus actual change in this setting are limited. The application of the SEIPS model to coach notes collected during the implementation of a structured behavioral intervention to improve the recognition and management of UTI in NHs generated unique insights into the work system and how staff attempted to implement changes. This study identified several factors that interfered with progression from planning to actual change. Future studies are needed to better understand how to best support change interventions in NHs.

**Trial registration:**

ClinicalTrials.gov, NCT03520010, Registered May 9, 2018.

**Supplementary Information:**

The online version contains supplementary material available at 10.1186/s43058-023-00535-y.

Contributions to the literature

- Use of existing artifacts (e.g., coach notes) is an alternative when a more robust evaluation is not feasible to obtain an understanding of how complex interventions are introduced and implemented in an organization.
- The Systems Engineering Initiative for Patient Safety (SEIPS) model provides a framework to assess how evidence-based interventions are implemented and how work systems components intersect to support implementation.
- Differentiating between planned versus actual changes could provide a greater understanding of how to design an intervention for successful implementation.

## Background

Implementation evaluations generate knowledge about how complex interventions are introduced and supported within an organization and may further help to understand why they succeed or fail. The focal point of such evaluations is on the activities or unique pathways that staff follow to achieve the intervention outcomes [1–5]. However, these evaluations do not commonly evaluate the unique pathways taken during intervention implementation [4, 6, 7]. A lack of understanding about which program elements are effective across multiple contexts and why they are effective makes it difficult to replicate the implementation of complex interventions [2, 7]. As such, efforts to address this gap is essential for the continued advancement of implementation research.

Efforts to conduct an implementation evaluation are equally complex. One approach is to examine how the intervention was introduced into the organization from multiple viewpoints. For example, the use of qualitative staff interviews and direct observations allows researchers to experience firsthand how the intervention is being used in the organization and interview stakeholders responsible for implementation efforts [8–10]. Other implementation evaluations often integrate qualitative and quantitative data to determine the efficacy of the intervention and the associated implementation strategies [11–13]. However, robust implementation evaluations involving direct observations and interviews with stakeholders as well as analysis and synthesis of a wide array of data elements are expensive and time consuming. Less resource-intensive evaluations may still provide useful information from study artifacts about the implementation process and its outcomes when more robust evaluations are not feasible [2]. One such artifact is coach notes, a written summary of the coach interactions with an organization. However, it is not clear if these notes are a routine part of an implementation analysis [14].

Herein, we describe an example of a less resourced implementation evaluation that focuses on how nursing home (NH) staff implemented an evidence-based urinary tract infection (UTI) recognition and management improvement toolkit (hereafter referred to as the “UTI toolkit”) [15]. Specifically, we conducted a work systems analysis with coach notes generated during the first 10 months of this study to better understand how staff moved from planning to effecting changes to implement the UTI toolkit in their respective NHs. While this approach is not meant to replace a full implementation evaluation, a coach note analysis offers important details on the various ways the study toolkit was implemented, provides an understanding of the work systems associated with planned versus actual changes during intervention implementation, and further illuminates how the organizational context impacted the overall success of implementation rollout.

## Methods

### Study setting

**Fig. 1:**
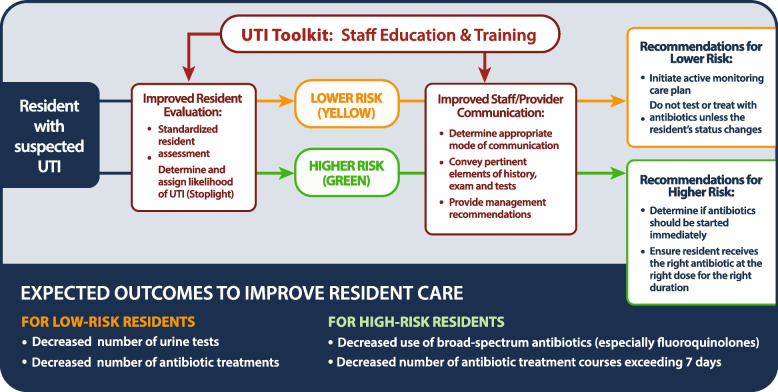
Wisconsin UTI improvement toolkit logic model. From: Effect of a Standard vs Enhanced Implementation Strategy to Improve Antibiotic Prescribing in Nursing Homes: A Trial Protocol of the Improving Management of Urinary Tract Infections in Nursing Institutions Through Facilitated Implementation (IMUNIFI) Study. JAMA Netw Open. 2019;2(9):e199526. doi:10.1001/jamanetworkopen.2019.9526

Data was collected as part of the IMUNIFI: Improving Management of UTIs in Nursing Institutions Through Facilitated Implementation (IMUNIFI) study, a cluster randomized trial examining the effectiveness of the UTI improvement toolkit to improve diagnosis and management of UTIs in 30 Wisconsin NHs [15]. The UTI toolkit is a multi-component resource that targets NH staff and clinician behaviors around the recognition and management of UTIs (Fig. 1). The toolkit is organized into five modules comprised of videos, slide sets, and handouts for clinicians, staff, and families (see Table 1). It was anticipated that NHs achieving these behavioral objectives would decrease the number of urine tests ordered, antibiotic treatments initiated, and treatment courses exceeding seven days as well as reduced use of broad-spectrum antibiotics.

**Table 1:** Overview of the UTI toolkit

Module name and sections	Objectives
**Module 1: Overview and rationale**	
• Overview • Clinical rationale • Regulatory rationale	• Provide an overview of the toolkit • Explain why antibiotic stewardship matters from clinical and regulatory perspectives • Educate providers, nursing staff, and family members of residents on appropriate management of UTIs
**Module 2: How to prevent catheter-associated urinary tract infection (CAUTI)**	
• Background and risk factors • Appropriate indications for indwelling catheter use • Indwelling catheter insertion and maintenance	• Provide guidance on appropriate use and management of indwelling urinary catheters • Provide guidance on how to properly collect a urine specimen from resident with a urinary catheter
**Module 3: When to test a urine specimen**	
• What is a urinary tract infection (UTI)? • When to submit a urine specimen for testing? • Case studies • Suggested educational plan	• Provide guidance on how reliably stratify residents into low and high risk of UTI • Increase nursing staff comfort with communicating assessment findings to providers and making recommendations for actions based on UTI risk • Provide guidance on how to perform active monitoring for residents with a low risk of UTI
**Module 4: When and how to treat a UTI**	
• When to treat? • How to treat? • How to modify?	• Provide the rationale and benefits of active monitoring • Provide guidance on antibiotic selection, dosage, and duration for treating a UTI • Provide rationale and guidance for performing a post-prescribing antibiotic timeout
**Module 5: Organizational tools**	
• Overview of quality improvement (QI) and how to lead change in the organization • The importance of tracking and reporting data for organizational QI • Sustainability of organizational change	• Provide guidance for assembling an improvement team • Provide examples of tools for use in the NH to help change frontline staff and provider behavior • Explain how data tracking and reporting can be used for organizational quality improvement • Discuss sustainability and the importance of developing a sustain plan

To learn more about the toolkit, please visit https://crc.chsra.wisc.edu/uti-toolkit/index.php

Participating NHs were recruited by email using a Wisconsin Department of Health Services listserv. NHs with more than a 50-bed capacity that expressed initial interest in study participation were required to demonstrate an ability to consistently submit self-reported data to a study website for three sequential months before enrolling in the study. Thirty NHs were randomized to either a usual implementation (control) group or enhanced implementation (intervention) group. NHs randomized to the enhanced implementation arm (*n* = 12) participated in a 1-day kickoff meeting and had access to the web-based toolkit, were assigned a clinical coach (a registered nurse or nurse practitioner with long-term care (LTC) experience), participated in peer-to-peer educational activities, and received peer comparison feedback reports. NHs randomized to the usual implementation group (*n* = 18) participated in a half-day kickoff meeting and had access to the web-based toolkit but did not receive other implementation resources. One NH dropped from the enhanced implementation group prior to implementation and was replaced with a similar NH from the usual care implementation group.

### Coach calls

Coaches assigned to NHs in the enhanced implementation arm provided external facilitation through bi-weekly calls with the NH designated internal champion—most often the NH infection preventionist or director of nursing. Coach interactions focused on supporting and encouraging teams to harness skills and resources toward the achievement of systemic change and improvement [16]. Initial calls reviewed current practices around the diagnosis and management of UTI as well as identification of opportunities to integrate the UTI toolkit into the NH’s existing work system. Subsequent calls reinforced important aspects of the implementation process, including (1) establishing a change team; (2) identifying barriers and facilitators to change; (3) reviewing and interpreting primary outcomes feedback reports; (4) identifying and prioritizing future change efforts; and (5) developing a plan to sustain improvement efforts.

We developed a standard coach template (hereafter, referred to as “coach notes”) to structure calls with each NH champion and record key aspects of the NH’s progress towards implementing the UTI improvement toolkit (Additional file 1). During the coach calls, coach notes were used to (a) review efforts by the NH to monitor data focused on UTI culture and treatment rates; (b) discuss facilitators and barriers encountered with implementation of the toolkit components; and (c) develop action plans to overcome identified barriers. The note also included general information about the call (date, NH name, participants, and duration). These notes, which captured the conversational essence of the interactions between the coach and NH champion regarding planned versus actual changes, were the data source for this analysis. In addition, coaches communicated to ensure that recommendations and practices were consistent across NHs and coach check-ins regularly occurred at research meetings. However, direct coach call observations or routine coach note reviews for completeness or depth did not occur.

### Guiding framework

A hybrid deductive-inductive approach, based on the Systems Engineering Initiative for Patient Safety (SEIPS) model [17], was used to analyze the coach notes documented from June 2019 to March 2020 (*n* = 148). The SEIPS model recognizes the work system complexity and the interconnectedness of tools and technology, tasks, person, physical environment, and organization used to support implementation of work system changes. Furthermore, the SEIPS model [1, 18–22] has been used to examine the work processes associated with antibiotic prescribing in LTC.

In the context of this analysis, we wanted to differentiate between planned versus actual changes associated with the implementation of the UTI toolkit in the NHs assigned to the enhanced implementation strategy. A planned change, defined as a discussion of ideas or plans to introduce elements of the toolkit in the NH but not the actual implementation, focused on how to implement the UTI toolkit in the NHs. In other words, a planned change represented the champions’ vision of how to roll-out the UTI toolkit. Since the implementation process may not have occurred as planned, actual changes represented the variability of the changes undertaken by the NHs to implement the UTI toolkit. In this study, actual change is defined as a discussion of how elements of the toolkit were implemented and used in the NH or conversations about how the toolkit improved care. Specifically, an actual change was identified as being present if the study facility champion performed specific tasks (e.g., staff training) to introduce the UTI toolkit in the NH.

### Data coding and analysis

We began our analyses by individually identifying themes related to the SEIPS 2.0 framework in the first two coach notes of each intervention NH (*n* = 24 total coach notes). We reviewed our themes to create agreed upon a priori codes and developed definitions for each code (Additional file 2). MaxQDA (2020, VERBI GmbH, Berlin, Germany) was used for coding. To further improve our codebook, we analyzed coach notes from two intervention NHs (*n* = 13 total coach notes) with our initial codebook to determine intercoder reliability (NH 1 k = 0.80, NH 2 k = 0.75). We analyzed the remaining coach notes individually, with 10% of the remaining notes being double coded (*k* = 0.85). For each coded segment, we included the number of codes per segment, the assigned coach, and the coach call to assign the coded segment to an intervention month.

**Fig. 2:**
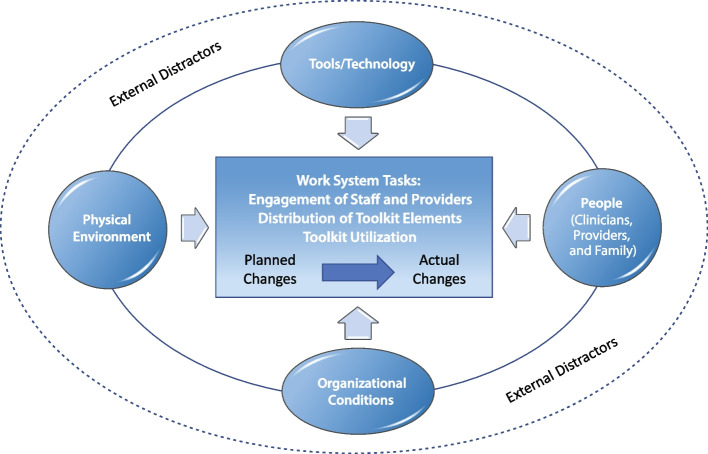
Transitioning from planned to actual changes in the context of the SEIPS model

After all coach notes were coded, we used codes and themes from 81% of the available coach notes. Twenty-six (19%) of coach notes were excluded because no segments were coded related to toolkit implementation or evidence of toolkit rollout plans. Within our final sample, we reviewed the associated quotes and identified three action domains that arose from coding related to (1) engagement of staff and providers, (2) distribution of toolkit elements, and (3) toolkit use (Table 2). Definitions of the action domains are in Additional file 3. Coach note segments within each action domain were coded as planned versus actual changes (Fig. 2). We then used the SEIPS model to explore how potential facilitators and barriers to the NH implementation efforts were affected by people, tools and technology, physical environment, and organizational conditions as well as external distractors as these efforts moved from planned to actual changes.

**Table 2:** Work task associated with the implementation of the UTI toolkit in nursing homes

Work task category	Work task category definition
Engagement of staff and providers (direct)	Inter-personal efforts to educate and/or train staff or providers about how to use the specific elements of the UTI toolkit (e.g., stoplight, watch and wait, provider clinical tool in module 4)
Distribution of toolkit elements (indirect)	Efforts that involved the distribution or display of specific materials from the UTI toolkit (e.g., posters, letters to providers, laminated cards)
Use of the toolkit	Details of how the NH staff was using toolkit elements (e.g., stoplight or scripts) as a part of their daily clinical care

This project was approved as quality improvement by the UW-Madison Health Science Institutional Review Board. The Standards for Reporting Qualitative Research (SRQR) checklist was used for this manuscript (Additional file 4).

## Results

The average size of the 12 participating NHs was approximately 80 beds, ranging from 50 to 99 beds. The NHs operated as non-profit (8 NHs), government run (2 NHs), or for profit (2 NHs) facilities. Half (50%) of the NHs were in rural counties. A total of 692 codes were identified in 369 different coach note segments with an average of 1.9 codes per coach note segment (Table 3). Most of the codes were related to the direct engagement of staff and providers (*n* = 373) or the indirect distribution of toolkit elements (*n* = 246). Planned changes were the focus of 62% of the total coach note segments. However, 96% of the conversations about toolkit use with NH champions focused on actual changes.

**Table 3:** Summary of coach segment coding by system perspective and work task

	**System perspective for toolkit rollout plans and toolkit implementation**								
	**Total**			**Planned changes**			**Actual changes**		
**Code summary**	**Coded segments**	**Total number of codes**	**Average # of codes**	**Coded segments**	**Total number of codes**	**Average # of Codes**	**Coded segments**	**Total number of codes**	**Average # of Codes**
**Total number of coded segments and codes**	**369**	**692**	**1.88**	**203**	**429**	**2.11**	**166**	**263**	**1.58**
Engagement of staff and providers (direct)	196	373	1.90	142	278	1.96	54	95	1.76
Distribution of toolkit elements (indirect)	116	246	2.12	58	148	2.55	58	98	1.69
Use of the toolkit	57	73	1.28	3	3	1.00	54	70	1.30

### Engagement of staff and providers

All NHs articulated plans for engaging their staff and providers. These activities consisted of active education and activities to raise provider awareness about and introduce staff to the UTI toolkit. Leadership support facilitated implementation. In NH 5, for example, the champion reported that the “Medical Director is fully on board and has reinforced the importance of stewardship at meetings with staff and providers”. All intervention NHs, at times, shared implementation plans with their coach that lacked specificity about how the UTI toolkit would be introduced. The lack of specific plans was most prevalent in four of the study NHs.

In NH2, the non-specificity of planned changes unfolded over four sequential coach calls. On the first call, the champion indicated that after talking to the director of nursing (DON) they “determined that they are too time consuming for the nurses meeting … [and]… made it mandatory that nurses review them”. However, a formal plan to evaluate staff knowledge and comprehension of the materials did not exist. The second call attempted to address the role of the champion, but it was deferred. Instead, the champion met with the DON to “determine a formal plan for the next steps for rolling out the toolkit with the [Skilled Nursing Facility] staff”. The third call focused on that meeting where the champion reported that:

The change team met today. Used an [internal planning] sheet to lay out plan for IMUNIFI. There was good engagement as the team identified barrier and opportunities. They outlined the steps required to fully implement the tool kit and identified responsibility for components—the [Nursing Home Administrator], DON and Nurses all took some assignments. They used resources from the tool kit. Talked about timeline and sustainability. Consensus that they need to focus more on education especially around the active monitoring/surveillance tool.

On the final call, just 2 weeks later, the champion still reported on the lack of implementation progress on the planned change, stating:

She has not had a chance to check back with the two nurses who have joined the change team to see if they have had a chance to work on tasks from the last meeting. They were to see if it is possible for ECS (electronic medical record program) to create a template or structure for charting to prompt documentation around toolkit interventions.

These delays in implementing a planned change were often influenced by multiple internal barriers. One such barrier was champion instability (e.g., turnover) which was identified in 7 of 12 NHs. For example, NH2 “start[ed] developing a training and transition plan for the DON to become the champion”. In NH3, the situation was more complex:

[the champion] has been off on maternity leave, she has not had a chance to review the UTI toolkit, resources or videos from kick off meeting. She currently has a colleague entering data, but no one else to cover her position.

These barriers may have influenced intervention effectiveness in this NH. Despite these issues with planning, 8 of 12 facilities (67%) were able to describe discrete actions to engage their staff, while 7 out of 12 NHs (58%) were able to describe actual steps taken to engage providers.

Staff meetings, including monthly in-service or new staff orientation, were the primary mode used to engage nursing staff. These meetings, which represented an actual change, focused on ongoing staff education or new staff orientation centered on providing staff with a working knowledge of the UTI toolkit. For example, NH6 used a monthly staff meeting where they would be “presenting information about the toolkit. They have a plan to introduce the material one bullet point at information”. For these meetings (actual change) to be effective, the champion needed to identify the crucial materials to review with staff to provide a working knowledge of the UTI toolkit. Champions also reviewed toolkit components or introduced tools such as the stoplight, scripts, or case studies. Similarly, NH1 reported that the “rollout [which occurred in the first month] has been effective …[and]… nurses asking good questions”. In certain situations, one-on-one communication was needed to educate a single individual or staff working on a different shift. For example, the champion of NH4 expressed concerns the night shift did not always have a complete picture about how to effectively use the UTI toolkit. To address this knowledge gap, she “sat down with one of the night nurses and asked her to share the module 3 with the night shift staff”. It represents an actual change in NH4 to implement the UTI toolkit.

In contrast to staff engagement, provider engagement occurred more frequently in one-on-one meetings. These included in-person meetings with the medical director and physician groups or through informal interactions with providers during routine clinical care encounters. For example, in NH11, designated staff members were “monitoring orders and trying to get regular providers to do antibiotic time outs- they have been successful in addressing the process after the fact in house”. This represented an actual change in NH11. Providers were not always supportive of the process, and NH11 expressed the importance of “facilitat{ing} good communication and trust” to effectively engage providers. Asynchronous communication with provider letters and/or brochures was another actual change NHs employed to engage their providers. For example, the champion at NH5 sent out the “Provider letter and trifold Provider Prescribing brochure to on call and [Emergency Room] providers to encourage participation”. The champion in NH9 indicated that “signed letters for providers and families will be sent out by the end of this month [July 2019]”.

Provider engagement in NHs that were unable to move beyond a planned change was inhibited by a variety of staff-related barriers, including champion instability (*n* = 3) and unplanned medical director absence (*n* = 1) Without a champion in place, the NH made little progress on efforts to engage staff and providers. For example, in the first month, NH2 had “plans on meeting with the [Emergency Department] medical director to discuss the UTI toolkit and further education for ED providers”. Three months later, the same champion was still discussing a planned change to meet “with the clinical providers and nurse educator to prepare training for ED providers”. Similarly, in NH11, the Medical Director had identified a planned change to engage providers as evidenced by the following statement: “Reviewed at [Quality Assurance and Performance Improvement] meeting and Medical Director is on board. Medical Director is strategizing about how best to approach ER providers fear of sepsis/death”. Four months after the initial Quality Assurance and Performance Improvement meeting with the medical director, the champion of NH11 stated that the medical director had not been able to assist with provider feedback due to time constraints and planned to step away from the role. Thus, this planned change was not implemented.

Ongoing engagement was a crucial component to toolkit rollout success. NHs reported a high frequency of staff onboarding, and champions in some NHs articulated a need to engage in ongoing training activities to sustain UTI toolkit implementation. For example, the champion in NH4 felt that “in order to sustain the momentum, they will need to be doing ongoing education- and teaching new people, who come in”. The champion in NH2 considered, as a planned change, “putting the UTI toolkit on the agenda during orientation, for each new hire to review” because the orientation involves a skills day including a focus on UTI prevention and catheter care which might be a good place to provide education around the UTI toolkit. Over the course of the study, the ongoing training and engagement journey in NH2 transitioned from in-person to online education. During initial coach calls, the champion “used some of the slides from Module 1 as an intro, … covered synopsis of the toolkit, overview of the study, posted some posters, handed out info, requested that the nurses watch Module 3 Sect. 1 and 2”. Approximately 2 months into the implementation process, the champion shared plans to integrate the UTI toolkit training into an online training module with the coach. Within a month, the online training module related to the UTI toolkit was complete and “nursing staff have until the end of Oct to complete this training for current nurses and [Certified Nursing Assistant]’s… [and]… it will be assigned to new employees during orientation”. In other words, NH2 moved from a planned to an actual change related to staff education about the UTI toolkit.

### Distribution of toolkit elements

Each participating NH received materials related to the different UTI toolkit elements to distribute or display (Additional file 5). The materials targeted three distinct groups: family, providers, and staff. The movement from planning to actual change varied depending on the targeted group. For example, the champion in 6 of the 12 NHs described planned changes related to the family, but it was unclear if these changes were implemented. Actual changes in the other 6 NHs were immediate and direct actions involved the use of multiple tasks (e.g., letter in admission packet, posting information in nursing station) to educate family and staff. In NH 6, the champion described their approach as trying to “Educates the families by putting the family letter in their admission packets and with her LTC residents, she reintroduces the information at the care conferences that occur every 3 months”. The champion of NH10 used a similar method of distribution, “Families are supplied info as they are admitted, in their admission packet and at the 3-month [Minimum Data Set] family care conference. Social workers have been good team members in this process”.

In addition to family education, NHs distributed toolkit elements to staff. In NH5, these changes involved:

[Champion] provided IMUNIFI training as part of their general orientation … [and] with copies of all written handouts. … [Champion] also walked them through the tools and gave them a photocopy of the first page of the [Clinical Resource Center (CRC)] web link and promoted them to use it as a valuable resource.

In NH2, the DON took steps to integrate some tools into the medical record to make it available to all staff as they “…incorporate[d] the toolkit stoplight for suspected UTI and list[ed] assessment and action steps for active monitoring and intervention”. This automation allowed for information from the tools to be automatically fed into the to do list for the nurses on each shift.

As an actual change, NHs also distributed information about the toolkit to providers. Activities included sending the brochure to internal providers (i.e., the nurse practitioners and providers with primary resident responsibility) and external providers (i.e., on-call or emergency department providers). For example, NH3 placed “written info went into each of their folders at the facility. They were expecting it—facility has been working on antibiotic stewardship for a while”. Typically, these actual changes were implemented by the champion during the first two months of the study or within a short time (< 1 month) when a new provider onboarded. However, challenges with NH leadership effectively communicating about the toolkit to sub-specialty or on-call physicians existed. For example, the champion in NH6 indicated that “It is always the on-call providers that continue to defy the recommendations not because they don’t know about the guidelines but because they are covering the bases because they don’t know the patient”. When the coach inquired if using the scripts would help address this issue, the champion indicated that they were unsure.

Competing demands, personal leave, new mandates, and NH audits all contributed to intervention NHs becoming stuck in the planning phase for extended periods of time. Internal or external distractors occurred in 50% or 6 NHs and contributed to intervention NHs becoming stuck in the planning phase for extended periods of time, thus not being able to move from planned to actual change. For example, the champion in NH3 said “they had a complaint survey on Monday of this week—that went well”. However, these external distractions impacted staff enthusiasm for change. The champion of NH11 stated that “they have had their annual survey followed by a Federal Look Behind Survey in the last month. Staff are exhausted.” Some external barriers were compounded by internal staff shortages as reported by the champion in NH1 that they faced “large mandates coming down from corporate and [were] short staffed”. In NH8, the champion summarized their competing demands as “investigation, self-report, surveyor visit, significant staffing problems, DON working the floor multiple shifts”. In NH6, the electronic health record conversion from one vendor to another vendor represented an internal distraction. These distractions directly impacted the ability of a NH to actively engage in the implementation of the UTI toolkit.

The champion of NH10 experienced nearly all these challenges within the first few months of the intervention. Not only was she new to her role in the NH at the start of the IMUNIFI project in July 2019 but just 1 week into IMUNIFI she had a planned medical absence which impacted her ability to distribute any toolkit elements to staff. In August 2019, the study coach expressed “concerns because it seems like we have talked about so many things but there has been no progress. I do not thing [think] there has been lack of cooperation, but rather due to the fact that she just started this job…She is in survival mode at this point”. NH10 underwent a site survey during September 2019, further complicating rollout. By November 2019, the first champion had resigned, and a new champion was appointed. Despite previous challenges, the new champion was able to develop a plan and continue rollout of the IMUNIFI project in December 2019. NH10 was not the only NH to experience these issues, the champion of NH8 was also “covering 2 positions and responsible for day-to-day operations” and “really hasn’t had time to look at [toolkit items]”. Ultimately, the NH8 champion also resigned from her position and the NH formally withdrew from the study in October 2019.

### Use of the toolkit

**Fig. 3:**
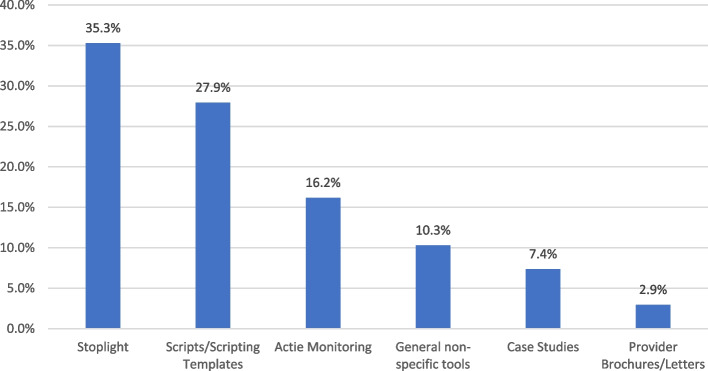
Staff use of clinical and provider tools from the UTI toolkit

The actual use of tools occurred quickly in most NHs with only 25% (*n* = 3) of the intervention NHs reporting getting stuck in the planning phase of how to introduce the tools. The stoplight, scripts and scripting templates, and active monitoring were the three most frequent clinical or provider tools (*n* = 68) used by NH staff (Fig. 3).

Scripts and scripting tools were introduced within the first three months of the implementation period, but actual use varied by NH. In the early stages of implementation, the champion of NH7 “posted the scripts and materials in each of the cubicles”; however, NH6 was waiting to introduce the scripts as part of the overall training for the UTI toolkit. In these examples, NH7 was engaged in actual change while NH6 was focused more on a planned change. In other NHs, staff used the stoplight in conjunction with active monitoring and the provider scripts. The champion of NH11 indicated that the staff “seem to be using the stop light and scripts when active monitoring is the most appropriate intervention”.

As an actual change, staff in 6 of the 12 NHs used active monitoring to evaluate residents for a UTI and potential antibiotic use. As the champion of NH11 indicated, “they [nurses] seem to be using the stop light and scripts when active monitoring is the most appropriate intervention”. While the champion of NH7 stated that they “continue to assess the nursing staffs’ use of the toolkit and possible times they encouraged active monitoring, as an intervention (if appropriate)”. Leadership support also was crucial in efforts to promote toolkit use. In NH5, it was reported that the “[medical director*]* has really been a good advocate for the use of tool kit materials … [and] gave an *in-service* to nursing staff recently reviewing the toolkit and reinforcing the need to focus on using alert charting form”.

## Discussion

Coaching is a discrete and effective implementation strategy [23]. A recent study found that coaching styles varied over time and was based on the focus of the organizational change efforts—preparation, implementation, and sustainment [24]. Our findings provide further evidence of the interactions between the external coach and the internal champion. In this study, the focus of the change efforts or the “thing” was the implementation of an evidence-based UTI toolkit in NHs [25]. The qualitative analysis of coach notes captured as part of the routine coaching call with NH staff provided insights into the “conversations” regarding the implementation of the UTI toolkit. Specifically, how staff leveraged knowledge [26, 27] and how the coaches’ implementation efforts (i.e., their action or activities) supported this knowledge acquisition [28, 29]. A similar approach has been used in other studies [30, 31]. Although the coach notes may not have been written as descriptively or in-depth as desired by implementation researchers, the knowledge gained can still be useful in our understanding of how implementation occurs at the organizational level. In this case, it could inform our understanding of work systems associated with planned and actual changes with a specific focus on the intervention adaptations associated with the implementation of the UTI toolkit in NHs.

Our analysis differentiated between planned change (e.g., the vision or ideas for rolling out the UTI toolkit) versus actual change (e.g., actions performed to introduce the UTI toolkit). Within the context of our study, the actual change, when it occurred, was during the active implementation period. Within the Framework for Reporting Adaptations and Modifications to Evidence-based Implementation Strategies (FRAME-IS), actual changes are related to staff training or engagement activities and on how the content (Toolkit tools) were introduced in the participating NHs [32, 33]. Our process expands on the concepts detailed adaption frameworks such as the FRAME-IS or the Dynamic Adoption Process [32–34]. Specifically, the coach note analysis identified planned changes that do not appear to be acted on by the champion in the participating NH. In addition, we focused on the relationship between the internal champion and the coach which does not appear to be captured as individuals who participated in the decision to modify an evidence-based practice. Future studies involving the analysis of coach notes should consider using an adaptation framework to better the actual process and reasons associated with planned versus actual changes.

In this study, we leveraged our experience in using the SEIPs model as a qualitative framework [35, 36] to examine work systems associated with planned versus actual changes. Specifically, we focused on efforts to engage staff and providers, distribute toolkit elements, and assess the actual NH use of the toolkit. In addition, we identified how organizational (champion instability) and environmental (e.g., regulatory visits) distractors as well as leadership support influenced how much time NHs engaged in planning change prior to transitioning to actual implementation of these changes. Thus, providing further evidence of the utility of the SEIPs model to evaluate work processes and process changes in implementation science.

Our analysis identified three reoccurring facilitators and barriers which often affected multiple aspects of implementation. Champion instability due to competing job demands, medical illness, or turnover was a common barrier identified in the current study and is consistent with other studies examining intervention implementation in other healthcare settings [37–41]. This instability either temporarily ceased (turnover) or slowed down (multiple roles) implementation efforts related to the rollout or implementation of the UTI toolkit. When this occurred, the transition from planned to actual changes was delayed by several months due to reliance on one individual (champion) to guide implementation efforts [42]. Similar delays were not seen in NHs with a stable champion. This is consistent with our findings where the champion had a crucial role in the successful rollout of the UTI toolkit. Specifically, the champion was able to communicate about the importance of the evidence-based practice, encourage formal and informal learning to promote implementation efforts, and perceive that they have organizational support to fulfill this role [43–46].

Ongoing and consistent education on the UTI toolkit is a critical component to sustain antibiotic stewardship programs [47, 48]. NH champions in our study addressed ongoing educational efforts by incorporating the trainings related to the UTI toolkit into new staff orientation meetings and by continuously allowing opportunities in monthly staffing meetings to discuss the UTI toolkit and the associated implementation efforts. One NH took this process a step further by working with their electronic health record (EHR) vendor to incorporate elements of the UTI toolkit into the EHR. This approach allowed staff to easily access tools when providing care.

External distractors also influenced implementation efforts [49]. In our project, these external distractions were associated with annual surveys or focused efforts to complete existing studies before implementing the UTI toolkit. External influences and competing demands stretched NH staff to their limits, making it difficult to successfully rollout the UTI toolkit within NHs. This is consistent with the literature which suggest that addressing daily clinical needs (i.e., “fires”) impacts implementation and hinders sustainability [47].

Leadership support affects implementation success in NHs [50, 51]. We experienced a similar situation in our study where 4 of the 12 NHs discussed with their coach how leadership supported the rollout of the UTI toolkit. How leadership support was offered varied across NHs. An example of actual change in support of the UTI toolkit rollout occurred in one NH where the medical director used the provider and family tools to communicate about the importance of UTI antibiotic stewardship in the NH. Conversely, in another NH, the support offered by the medical director was conceptual in that they discussed plans on how to engage difficult to approach providers. However, the coach notes indicated a failure to move from planned to actual changes 4 months after the initial meeting.

### Limitations

Our study had several limitations. NHs that were overwhelmed may have had fewer coach calls and the information captured in the coach notes varied by NH. Therefore, our understanding of implementation of the UTI toolkit in NHs with fewer calls or incomplete information was limited. Our study was based on the analysis of coach notes; however, our sample excluded 19% of coach notes. These segments were excluded because they were not coded as being associated with toolkit implementation or evidence of toolkit rollout plans.

Our analysis was based solely on self-reported coach notes. The quality or depth of content included on the coach notes was not examined. As such, it is possible that some coaches wrote more on their notes versus others and the content analysis of the coach notes may have missed key discussions of planned versus actual changes. In addition, we did not use interviews with NH staff or coaches or direct observation (e.g., listening to or recording the coach calls) to understand how the UTI toolkit was implemented in the NH. This approach, typically associated with a more expensive ethnographic analysis, may have provided a more robust understanding of the planned versus actual changes associated with implementation of the UTI toolkit. The absence of direct observation of coaching calls, structure fidelity checks, or efforts to ensure content completeness and depth in the coach notes may also have impacted our understanding of actual versus planned changes.

Our study did not examine the actual degree of the coach-champion interactions on the calls individually or over time. It is possible that some NH champions required more direct coach support while other NH champions implemented the UTI toolkit with limited coach support. The use of recordings or transcribed notes of the coach sessions may provide a better understanding of the interactions and its impact on implementation of an evidence- based practice.

## Conclusions

Use of existing data sources offers a unique opportunity to explore the implementation of a complex intervention when sufficient resources and time for a full-scale implementation evaluation is not feasible. This project may inform future researchers’ efforts to develop unique and cost-effective ways to conduct an implementation evaluation. Since coaching is a proven implementation strategy, the use of artifacts such as coach notes recorded at the time of the interaction between the coach and the champion provides an alternative low-cost implementation evaluation approach. The use of the SEIPs model to analyze coach notes provides a framework to evaluate what “goes on behind the curtain” and offers unique insights into the work systems necessary to implement evidence-based interventions in NHs. More importantly, we identified leadership support as an important element in the implementation of antibiotic stewardship programs in NHs especially when a NH is experiencing high staff turnover and external distractors.

Our study differentiated between planned versus actual changes. More importantly, we identified that NH work systems supporting implementation are influenced by leadership support, champion instability, and external environmental distractors. These efforts potentially help address a gap in perspectives within a clinical setting about how to best help researchers and staff evaluate the implementation of an evidence-based practice such as the UTI toolkit. Given the importance of antibiotic stewardship in NHs, more work on understanding how to implement and sustain antibiotic stewardship interventions in NHs is needed.

### Supplementary Information

**Additional file 1.** Templated Coach Note.

**Additional file 2.** IMUNIFI Coach Note Code Book Dictionary v. 5/7/2020.

**Additional file 3.** Theme Categories and Definitions.

**Additional file 4.** SRQR Checklist.

**Additional file 5.** UTI Toolkit Elements.

## Data Availability

The datasets used and/or analyzed during the current study are available from the corresponding author on reasonable request.

## Abbreviations

CRC: Clinical Resource Center
DON: Director of nursing
EHR: Electronic health record
IMUNIFI: Improving Management of UTIs in Nursing Institutions Through Facilitated Implementation
LTC: Long-term care
NH: Nursing homes
SEIPS: Systems Engineering Initiative for Patient Safety
UTI: Urinary tract infection
