# Supplementary material for: Moving behavioral interventions in nursing homes from planning to action: a work system evaluation of a urinary tract infection toolkit implementation
Source: Implement Sci Commun. 2023 Dec 12;4:156. doi: 10.1186/s43058-023-00535-y (PMC10714494; doi:10.1186/s43058-023-00535-y)
Supplement: Supplementary file 1 — Additional file 1. Templated Coach Note. [file 43058_2023_535_MOESM1_ESM.docx]

**Additional file 1**  Templated Coach Note

**Facility Name**

Facility Address

**Date**: **Last Call** **Participants:**

**Champion: Phone**: **Email:**

**# of Beds:**  **Location:** **Status:** (FP/NFP) **Urban/Rural Lab:**

**Medical Director: Pharmacy: Pharmacy Consultant:**

**Med Record Program:**  **How are Provider Visits Handled:**

**What is after hours call system Usual Inservice Format:**

**Medicare A # licensed beds—**

**Duration of Call** **Change in Critical Staff since last call**

Any variations, cancelled meetings, unusual circumstances etc since last coaching session.

**REVIEW OF DATA**

- Any problems entering data?
- Has data been entered in a timely fashion (by 15^th^ of previous month)? Add date last updated
- Any questions regarding reports – internal or benchmark?

Insert facility data reports since last coach session to review (cut and paste from CRC)

Urine Cultures

Antibiotic Starts

Average Days of Treatment

**IMPLEMENTATION**

Toolkit Implementation Plan?

Standardized COC assessment?

Staff using Stoplight to assess UTI risk?

Facilitators to Implementation of Assessment Plan

Frontline Staff

Providers

Administration

Barriers to Implementation of Assessment Plan

Frontline Staff

Providers

Administration

Competing Demands**:**

**Communication – Staff to Provider**

Implementation Plan for Roll out to Providers?

Using Case Studies/Script?

Using appropriate mode of communication?

Conveying pertinent information?

Providing recommendations to provider?

Facilitators for communication

Frontline Staff.

Providers

Administration

Barriers to communication

Frontline Staff

Providers

Administration

Competing Demands

**Appropriate Provider Action**

Low risk

Recommending active monitoring if appropriate?

Not testing or treating unless change in status?

High risk

Determining if warning signs warrant immediate AB or higher level of care

Correct AB/Correct dose/Correct duration

Facilitators to Appropriate Provider Action

Frontline Staff

Providers

Administration

Barriers to Appropriate Provider Action

Frontline Staff

Providers

Administration

Competing Demands—

**Action Plan from Previous Call**

Progress:

Barriers:

**SUMMARY**

**Call Summary:**

**Action Plan**

Details for next call

Date:

Time:

**Contact Information**

**Coach: Name Phone Email**

**Champion: Name Phone Email**
