# Supplementary material for: Moving behavioral interventions in nursing homes from planning to action: a work system evaluation of a urinary tract infection toolkit implementation
Source: Implement Sci Commun. 2023 Dec 12;4:156. doi: 10.1186/s43058-023-00535-y (PMC10714494; doi:10.1186/s43058-023-00535-y)
Supplement: Supplementary file 2 — Additional file 2. IMUNIFI Coach Note Code Book Dictionary v. 5/7/2020. [file 43058_2023_535_MOESM2_ESM.docx]

**Additional file 2** IMUNIFI Coach Note Code Book Dictionary v. 5/7/2020

| **#** | **Code** | **Definition** |
| --- | --- | --- |
| 1 | Leadership Support | Specific mention of support by NH leadership for the toolkit implementation efforts |
| 2 | Coach prompting review of toolkit or suggesting implementing an aspect of the toolkit | Describing a coach's suggestion to the champion to review the toolkit |
| 3 | Coach suggestion | Describing a coach's suggestion to implement an aspect of the toolkit to address a barrier |
| 4 | Plans for data review and feedback | Mention of plans to use the data feedback reports or create a feedback mechanism but plans have not been implemented |
| 5 | Family resistance | Champion describing situations where resident family members have expressed resistance to the toolkit or modifying their prescribing behaviors |
| 6 | Staff resistance | Champion describing situations where staff have expressed resistance to the toolkit or modifying their behaviors |
| 7 | Champion not in place | Was no champion or champion was absent due to personal reasons (vacation, family health matters, illness, etc.) |
| 8 | Champion turnover | Coach indicates new champion in place learning their position |
| 9 | Data review and feedback | Efforts by the NH to review antibiotic prescribing data to understand why an ABX was ordered, understand ABX patterns, identify barriers to appropriate care, or a similar use of data review process |
| 10 | Barriers: Data entry | Describing barriers related to data entry on the CRC website |
| 11 | Provider engagement | Champion describes engaging with a provider to educate them on the toolkit |
| 12 | Pharmacist engagement | Champion describes engaging with a pharmacist to collect data or educate them on the toolkit |
| 13 | Provider resistance related to toolkit implementation | Champion describing situations where providers have expressed resistance to the toolkit or modifying their prescribing behaviors |
| 14 | Provider support for toolkit implementation | Champion describing situations where providers have expressed support for toolkit implementation |
| 15 | Lack of toolkit roll out plans | Describing the general sense that a champion does not have a plan in place to roll out elements of the toolkit |
| 16 | Toolkit roll out plans | Champion describing any type of plans to roll out elements of the toolkit but they have not been implemented |
| 17 | Staff turnover impacting implementation | Staff turnover negatively impacting the implementation of the UTI toolkit (related to staff education) |
| 18 | Staff overwhelmed | Champion describing staff being overwhelmed by other NH projects (other surveys, competing projects, high staff turnover, etc.) |
| 19 | Champion facing barriers to implementation | Describing the champion facing barriers to implementing the toolkit at their NH |
| 20 | Lack of champion engagement | Describing a situation where the champion has shown lack of engagement with toolkit implementation (has rescheduled a call with the coach, has not reviewed the toolkit prior to telephone calls, etc.) |
| 21 | Champion overwhelmed | Describing a situation where either the coach describes or the champion explicitly states that they are (champion is new to the role, champion has multiple competing duties within the NH, etc.) |
| 22 | Challenge to implement with certain populations | Champion describes difficulty implementing the toolkit with specific populations (older adults with dementia). |
| 23 | Current process in place at NH to address antibiotic prescribing | Champion describing current tools or processes in place at their NH to influence antibiotic prescribing (Loeb or McGeer criteria posters, etc.). |
| 24 | Family education | Champion describing instances of educating families utilizing toolkit |
| 25 | Staff engagement/ commitment | Champion describes staff willingness to engage with the toolkit |
| 26 | Positive results | Champion describing situations where the toolkit has had a positive impact on staff behavior (implementing watch and wait, utilizing brochure/ video to educate family) |
| 27 | Staff education | Champion describing instances of educating staff on toolkit or barriers to educating staff on toolkit |
| 28 | 1. Toolkit implementation | Describing the implementation of some element of the toolkit (hanging posters, watching videos, sending letters to providers) |
| 29 | Study withdrawal decision | Indication by the nursing home that they are withdrawing from the study |
| 30 | Engagement plans with coach | Coach note describes plans made by coach and champion for future engagement (frequency of meetings, discuss planned time off, etc.) |
| 31 | Input about IMUNIFI Components | Mention by the champion about the overall quality of an IMUNIFI component such as webinar, kick-off meeting, etc. |
