# Supplementary material for: Moving behavioral interventions in nursing homes from planning to action: a work system evaluation of a urinary tract infection toolkit implementation
Source: Implement Sci Commun. 2023 Dec 12;4:156. doi: 10.1186/s43058-023-00535-y (PMC10714494; doi:10.1186/s43058-023-00535-y)
Supplement: Supplementary file 3 — Additional file 3. Theme Categories and Definitions. [file 43058_2023_535_MOESM3_ESM.docx]

**Additional file 3** Theme Categories and Definitions

| Theme | Definition |
| --- | --- |
| **Engagement of staff and providers** |  |
| Plans for provider engagement | Plans of the champion to discuss the toolkit with provider(s) in person or encourage them to review a module |
| Plans for staff or provider education | Plans of the champion/ change team to educate the staff in either 1:1 meeting, group settings (scheduled in-service meetings, scheduled staff meetings, meetings scheduled specifically to review the toolkit), or in a virtual space (NH specific learning platform or watching module videos) |
| Actual provider engagement | Champion references continued meetings with providers who are resistant to toolkit implementation |
| Staff or provider education | Education that took place as either 1:1 meeting, in group settings (scheduled in-service meetings, scheduled staff meetings, meetings scheduled specifically to review the toolkit), or in a virtual space (NH specific learning platform or watching module videos) |
| **Distribution of toolkit elements** |  |
| Plans to display toolkit elements | Plans of the champion/change team to hang posters or stoplight tools in areas visible to staff |
| Plans to distribute toolkit elements | Plans of the champion/ change team to give brochures, provider letter, hard copies of toolkit, send links to toolkit, and share videos with staff, providers, and family |
| Toolkit elements displayed | Champion/change team hung posters or stoplight tools in areas visible to staff |
| Toolkit elements distributed | Pocket cards, scripts, hard copies of toolkit, links to toolkit, etc. were distributed to staff, providers, and families |
| **Toolkit use** |  |
| Actual use of toolkit items | Champion references staff utilizing specific toolkit items (scripts, stoplight, etc.) |
