# Supplementary material for: Moving behavioral interventions in nursing homes from planning to action: a work system evaluation of a urinary tract infection toolkit implementation
Source: Implement Sci Commun. 2023 Dec 12;4:156. doi: 10.1186/s43058-023-00535-y (PMC10714494; doi:10.1186/s43058-023-00535-y)
Supplement: Supplementary file 5 — Additional file 5. UTI Toolkit Elements. [file 43058_2023_535_MOESM5_ESM.docx]

**Additional file 5** UTI Toolkit Elements

| **Educational Item** | **Description** | **Type of Educational Material Provided** | | | |
| --- | --- | --- | --- | --- | --- |
|  |  | Magnet | 5 x 7 Card | 8.5 x 11 | Poster |
| Stoplight | A tool design to help clinical staff know when and when not to test for a UTI | 5 x 3 Fridge Magnet | Laminated One Side | Non Laminated 11 x 8.5 Pad |  |
| Active monitoring | A form that allows clinical staff to track signs and symptoms of an individual patient |  | Laminated Double Sided | Non Laminated Pad |  |
| When to test urine nursing tool | A flowchart that walks nurses through the design process to determine when to test versus active monitoring |  | 7 x 5 Laminated | 11 x 8.5 Double sided non laminated pad | 15 x 11 One-sided poster |
| Infographic on management of suspected UTI in NH | An overview of the management of UTIs in NH including common myths and facts and when and when not to test |  |  | 8.5 x 11 two sided non laminated | 11 x 17 one sided two page poster |
| Provider trifold brochure | An educational brochure for providers about managing suspected UTI in NH |  |  | Tri-fold 11 x 8.5 Laminated Double Sided Brochure |  |
| Family education letter | A templated letter for use by the NH to explain UTI treatment philosophy to resident families |  |  | 8.5 x 11 one sided non laminated |  |
| Family education brochure | An educational brochure for families about the NH approach to UTI treatment |  |  | 8.5 x 11 one sided non laminated |  |
